# Hepatic estrogen receptor α is critical for regulation of gluconeogenesis and lipid metabolism in males

**DOI:** 10.1038/s41598-017-01937-4

**Published:** 2017-05-10

**Authors:** Shuiqing Qiu, Juliana Torrens Vazquez, Erin Boulger, Haiyun Liu, Ping Xue, Mehboob Ali Hussain, Andrew Wolfe

**Affiliations:** 1 Division of Metabolism and Pediatric Endocrinology, Departments of Medicine, Pediatrics, Biological Chemistry and Physiology, Johns Hopkins University School of Medicine, Baltimore, MD USA; 2 School of Medicine, Ponce Health Sciences University, Ponce, PR USA; 3 School of Engineering, Johns Hopkins University, Baltimore, MD USA; 4 Department of Dermatology, Johns Hopkins University School of Medicine, Baltimore, MD USA

## Abstract

Impaired estrogens action is associated with features of the metabolic syndrome in animal models and humans. We sought to determine whether disruption of hepatic estrogens action in adult male mice could recapitulate aspects of the metabolic syndrome to understand the mechanistic basis for the phenotype. We found 17β-estradiol (E_2_) inhibited hepatic gluconeogenic genes such as phosphoenolpyruvate carboxykinase 1 (*Pck*-*1*) and glucose 6-phosphatase (*G6Pase*) and this effect was absent in mice lacking liver estrogen receptor α (*Esr1*) (LERKO mice). Male LERKO mice displayed elevated hepatic gluconeogenic activity and fasting hyperglycemia. We also observed increased liver lipid deposits and triglyceride levels in male LERKO mice, resulting from increased hepatic lipogenesis as reflected by increased mRNA levels of fatty acid synthase (*Fas*) and acetyl-CoA carboxylase (*Acc1*). ChIP assay demonstrated estradiol (E_2_) induced ESR1 binding to *Pck*-*1*, *G6Pase*, *Fas* and *Acc1* promoters. Metabolic phenotyping demonstrated both basal metabolic rate and feeding were lower for the LERKO mice as compared to Controls. Furthermore, the respiratory exchange rate was significantly lower in LERKO mice than in Controls, suggesting an increase in lipid oxidation. Our data indicate that hepatic E_2_/ESR1 signaling plays a key role in the maintenance of gluconeogenesis and lipid metabolism in males.

## Introduction

Beyond its canonical role in reproductive development and function estrogens also plays a role in regulating non-reproductive systems such as immune function^1, 2^, growth^3, 4^, neuronal function^5–8^ and metabolism^9–11^. Mice with aromatase deficiency and inability to synthesize estrogens exhibit disrupted metabolic function^12^, and there are dramatic metabolic changes that occur with the normal changes in reproductive status across the lifespan, including during puberty^13^, the menstrual cycle^14^ and menopause^15^. Additionally hypogonadism in men is associated with increased risk of type 2 diabetes and metabolic syndrome^16, 17^. Therefore, an understanding of the mechanisms underlying gonadal steroid regulation of glucose and lipid metabolism is required.

Cellular 17β-estradiol (E_2_) signaling is mediated primarily via the nuclear hormone receptors estrogen receptor (ER) alpha (ESR1) and ER beta (ESR2), although recent findings have demonstrated E_2_ action via the cell surface G-protein coupled receptor, GPER^18^. ESR1 and GPER are the major estrogen receptors expressed in the liver, with ESR1 being much more abundant than GPER^18^. Impaired ESR1 function is associated with obesity and metabolic dysfunction in humans^19, 20^ and rodents^21–24^. However, the mechanisms underlying these phenotypes still remain largely elusive.

Hepatic glucose production is critical for maintaining normoglycemia in the fasting state, providing fuel for the brain, renal medulla, and red blood cells. Unregulated hepatic glucose production (HPG) is a feature of diabetes mellitus and contributes to fasting hyperglycemia^25^. Hepatic gluconeogenesis is regulated by transcriptional modulation of the key gluconeogenic enzymes phosphoenolpyruvate carboxykinase (*Pck1*) and glucose-6-phosphatase (*G6Pase*).

The liver also plays an important role in lipid metabolism. Hepatic triglyceride synthesis is the sum of two main processes: the synthesis of fatty acids (*de novo* lipogenesis, DNL) and esterification of fatty acids into fatty-acid glyceride species. Acetyl CoA carboxylase (ACC) and fatty acid synthase (FAS) play important roles in DNL. Knockdown of ACC, which is crucial in the regulation of DNL and lipid oxidation, reduces liver triglyceride and diacylglycerol content and protects mice from lipid-induced hepatic insulin resistance^26^. Treating high-fructose diet-fed db/db mice with FAS inhibitor plantensimycin reduces hepatic lipid accumulation and hepatic fatty acid oxidation^27^. These data highlight the essential roles of both ACC and FAS in DNL and metabolic homeostasis.

The aim of the current study was to mechanistically explore the role of hepatic ESR1 in male mice in regulating glucose and lipid metabolism. A sexual dimorphism was reported in the aromatase knockout (ArKO) mouse model with males exhibiting impaired hepatic insulin sensitivity, glucose and pyruvate intolerance^28^, while females did not exhibit these metabolic features. Therefore, we sought to determine the role of hepatic ESR1 signaling in regulating hepatic function in males by acutely knocking out ESR1 specifically in the liver (LERKO mice). We observed that acute liver-specific disruption of ESR1 increases the expression of key gluconeogenic and lipogenic enzymes which also resulted in increased gluconeogenesis and dyslipidemia. These results suggest that ESR1 is critical for the regulation of gluconeogenesis and lipid metabolism, and that alteration in ESR1 expression in the liver could contribute to dysregulation of glucose homeostasis and dyslipidemia.

## Results

### LERKO mice showed liver specific knockdown of *Esr1*

**Figure 1:**
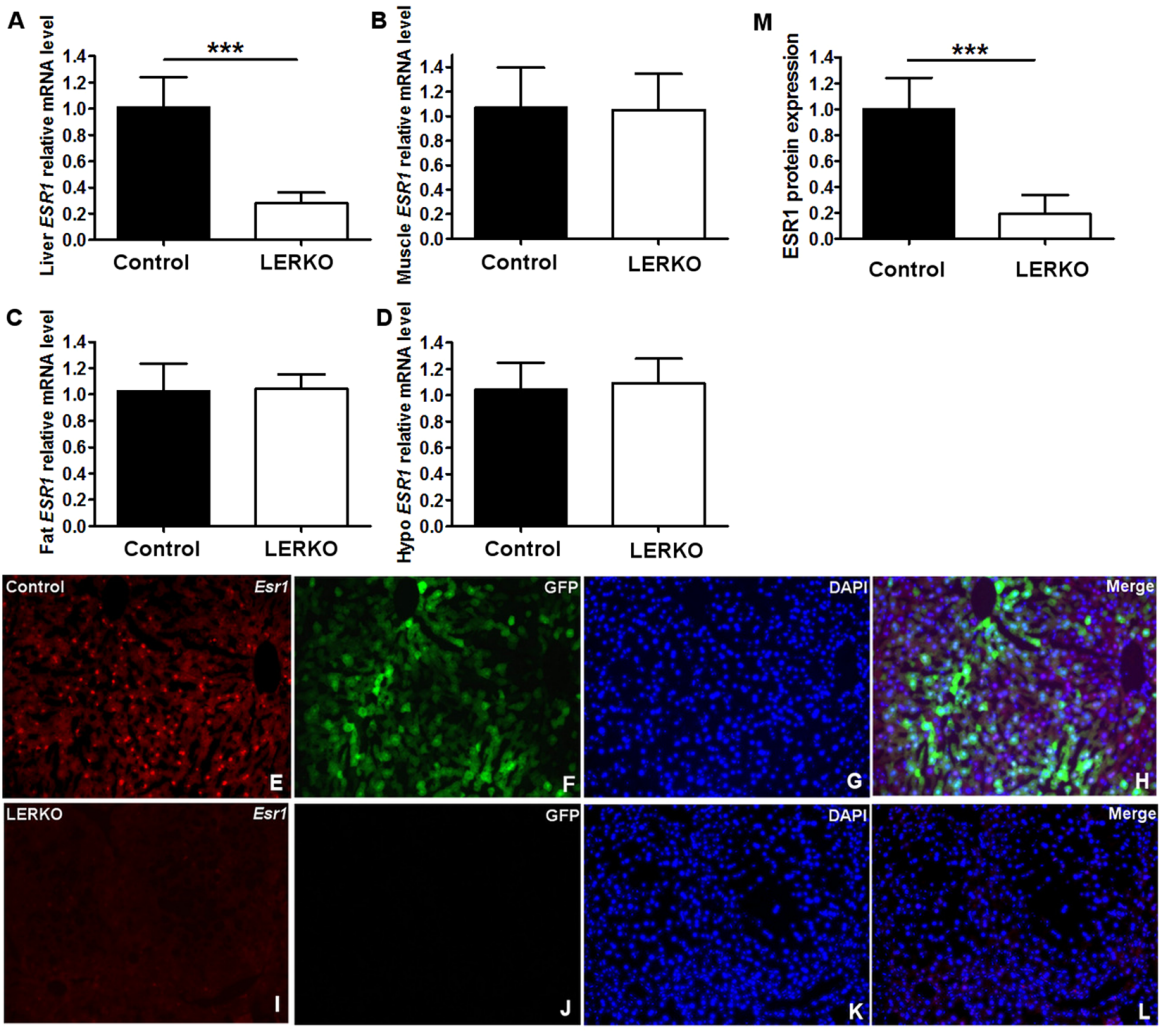
Knock down of hepatic ESR1 in the LERKO mice. (**A**–**D**) mRNA levels of ESR1 in liver, muscle, fat and hypothalamus measured by q-RT-PCR. (**E–L**) immunofluorescence staining of hepatic ESR1 from Control (**E**–**H**) and LERKO mice liver (**I**–**L**). (**E**,**I**) ESR1 staining. (**F**,**J**) GFP fluorescence. (**G**,**K**) DAPI staining. (**H**,**L**) merged images. (**M**) ImageJ quantification of immunofluorescence staining (n = 6). Data are expressed as the means ± SD, *p < 0.05 versus Control, **p < 0.01 versus Control, ***p < 0.001 versus Control.

To assess whether *Esr1* was knocked down specifically in the liver, *Esr1* mRNA levels were examined by q-RT-PCR. LERKO mice exhibited significantly reduced *Esr1* mRNA levels specifically in the liver, and not in adipose tissue, muscle tissue, or hypothalamus (Fig. 1A–D). Immunofluorescence showed that ESR1 protein was absent in liver sections of LERKO mice (Fig. 1I) but present in Controls, (Fig. 1E), AAV8-GFP Control vector injected mice give a visual confirmation of the uniform distribution of viral integration (Fig. 1F).

### LERKO mice demonstrated elevated gluconeogenesis relative to Control mice

**Figure 2:**
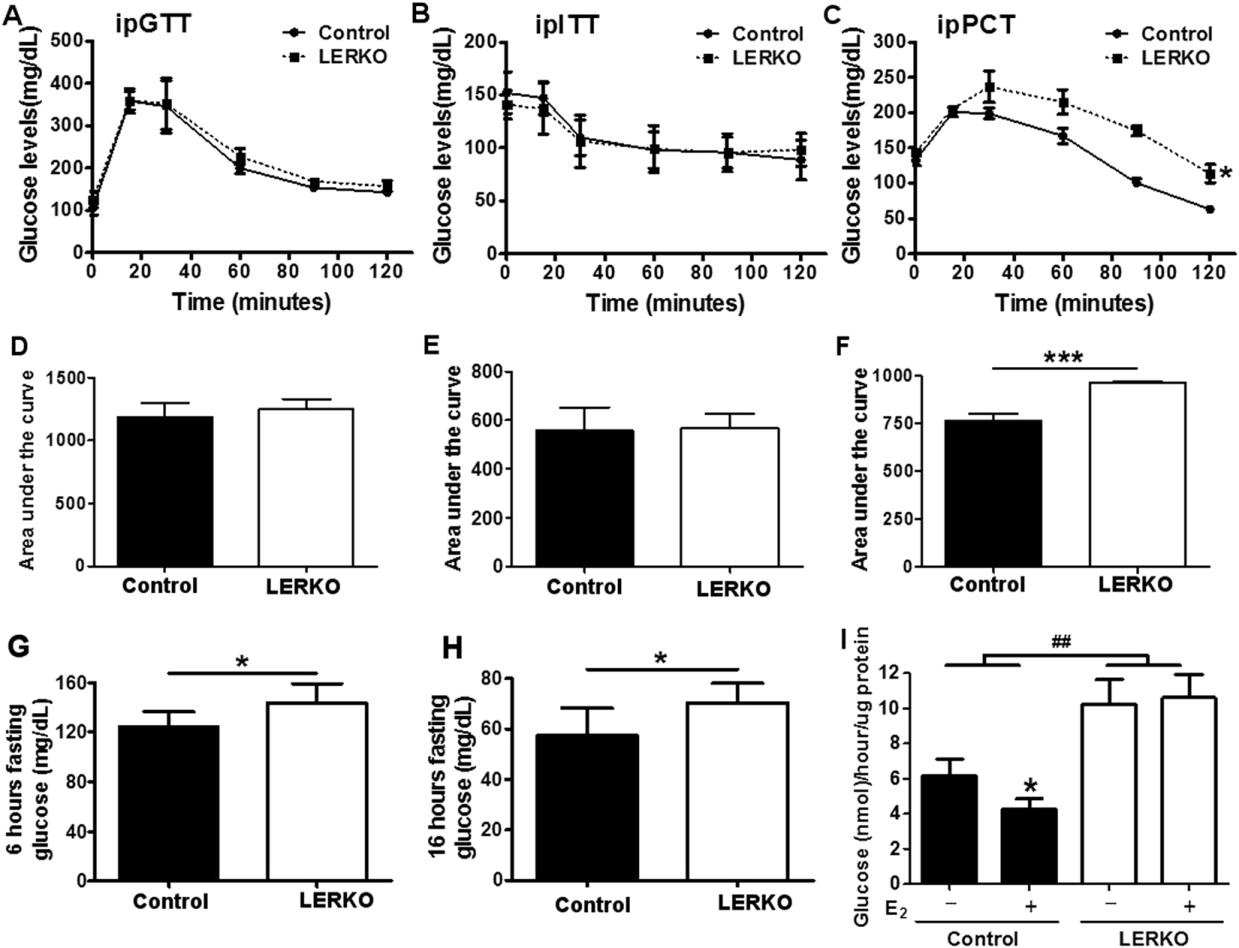
Glucose, insulin and pyruvate tolerance tests. (**A**) Glucose tolerance test (GTT) was performed on Control (circle symbols) and LERKO (square symbols) mice. Glucose (2 g/kg body weight) was administrated by intraperitoneal injection after overnight fasting. (**D**) Area under the curve of the GTT displayed in (**A**). (**B**) Insulin tolerance test (ITT) was performed on Control (circle symbols) and LERKO (square symbols) mice. Insulin (0.3 U/kg body weight) was administrated by intraperitoneal injection after 7 hours fasting. (**E**) Area under the curve of the ITT displayed in (**B**). (**C**) Pyruvate challenge test (PCT) was performed on Control (circle symbols) and LERKO (square symbols) mice. Pyruvate (2 g/kg body weight) was administrated by intraperitoneal injection after 6 hours fasting. (**F**) Area under the curve of the PCT displayed in (**C**). *p < 0.05 versus Control. (**G**–**H**) 6 hours and 16 hours fasting glucose of Control and LERKO mice. *p < 0.05, ***p < 0.001 versus Control (Fig. 2A–H). (**I**) Glucose production assays were conducted in primary hepatocytes from Control and LERKO mice; cells were subjected to 4 hours of serum starvation before the addition of E_2_ for 12 hours. The experiments were performed 1–3 weeks after virus injection. The data are expressed as the means ± SD, *p < 0.05, vehicle versus E_2_ treatment, ^##^ *p* < 0.01 Control versus LERKO (Fig. 2I).

LERKO and Control mice displayed similar glycemic excursions and glucose AUC during ipGTT (Fig. 2A and D). Similarly, ipITT was similar in LERKO and Control mice. (Fig. 2B and E). However, LERKO mice showed a robust elevation of gluconeogenic capacity during intraperitoneal pyruvate challenge test as compared to Controls (*p < 0.05 Control vs. LERKO on chow diet; Fig. 2C and F). Additionally, 6 hour and 12 hour fasting blood glucose levels were higher in LERKO mice compared to Controls (Fig. 2G–H). To further support the observation that knocking down hepatic ESR1 resulted in increased hepatic glucose production (HGP), we directly measured glucose production in primary hepatocytes. Hepatocytes isolated from LERKO mice produced significantly more glucose *in vitro* than hepatocytes from Control mice (Fig. 2I). Importantly, *in vitro* E_2_ treatment reduced glucose production in hepatocytes isolated from Control mice but not from LERKO mice (Fig. 2I).

### E_2_ inhibits gluconeogenic genes through ESR1

**Figure 3:**
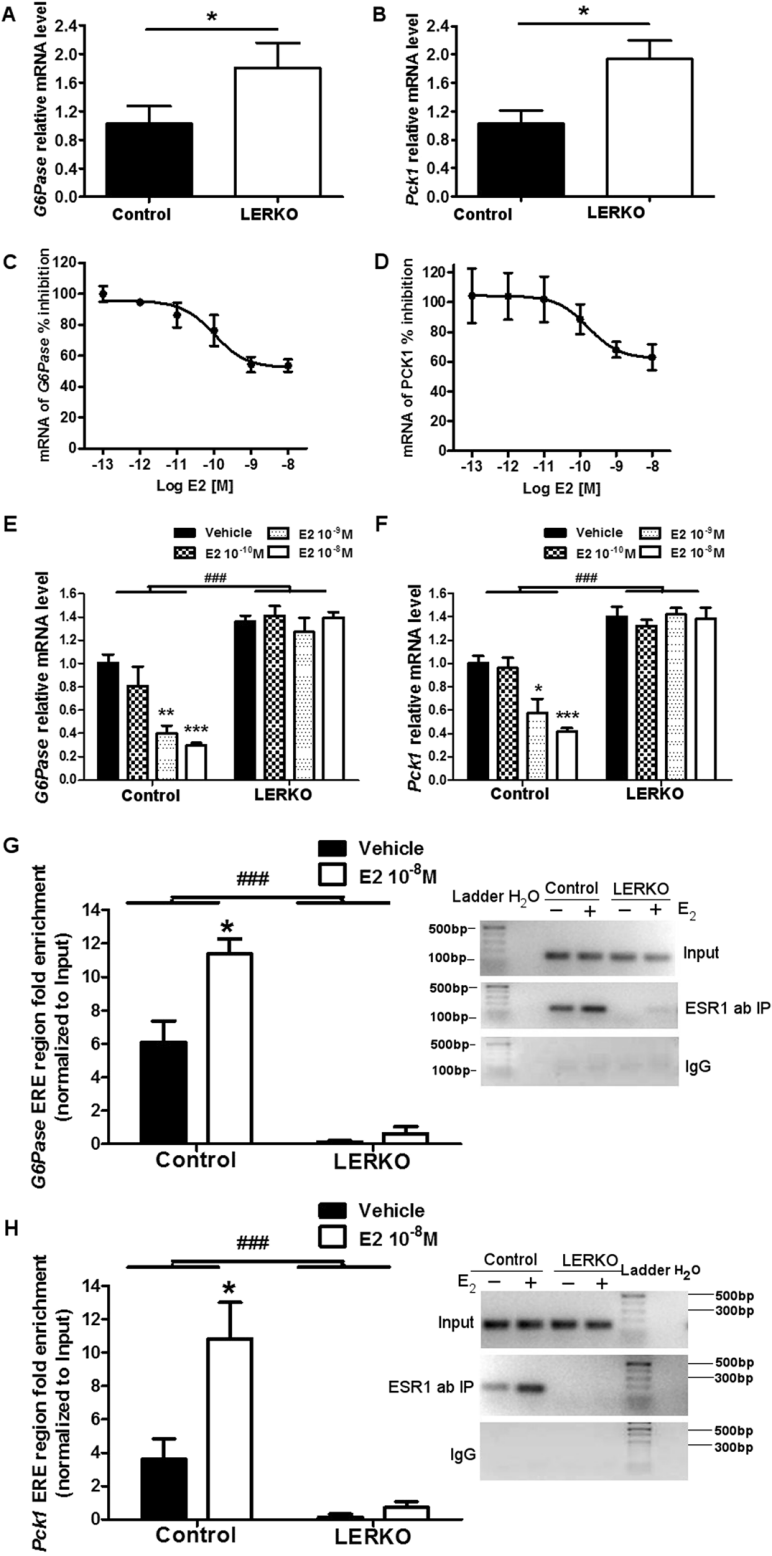
ESR1 inhibition of gluconeogenic gene expression is E_2_ dependent. (**A**,**B**) Hepatic mRNA levels of *G6Pase* and *Pck1* from Control mice and LERKO mice (n >= 6 per genotype). (**C**,**D**) Primary hepatocytes from Control mice were treated with vehicle or different doses of E_2_ (10^−12^ M, 10^−11^ M, 10^−10^ M, 10^−9^ M, 10^−8^ M), mRNA levels of *G6Pase* and *Pck1* were measured by q-RT-PCR. F-H, primary hepatocytes from Control and LERKO mice were treated with vehicle or different doses of E_2_ (10^−10^ M, 10^−9^ M, 10^−8^ M), mRNA levels of *G6Pase* and *Pck1* were measured by q-RT-PCR. (**E**,**F**) Primary hepatocytes from Control and LERKO mice were treated with vehicle or different doses of E_2_ (10^−10^ M, 10^−9^ M, 10^−8^ M), mRNA levels of *G6Pase* and *Pck1* were measured by q-RT-PCR.G-H, ChIP assay experiments were performed with liver tissues using antibody to ESR1, or with rabbit preimmune serum (IgG) and primers flanking the *G6Pase* (**G**) and *Pck1* (**H**) promoters. Real-time PCR data with an inset of a 1.5% agarose gel as a representative example. Results were normalized to input and shown as fold enrichment IgG from 3 independent ChIP experiments. The experiments were performed 2 weeks after virus injection. The data are expressed as the means ± SD, *p < 0.05 versus control, *p < 0.05 versus vehicle, ^###^ *p* < 0.001 versus control.

Gluconeogenesis is thought to be regulated at the transcriptional level^29^, thus mRNA levels of key gluconeogenic enzymes were examined by q-RT-PCR in Control and LERKO mice. Consistent with the results from the pyruvate challenge test (Fig. 2C), LERKO mice showed a dramatic increase of mRNA levels of phosphoenolpyruvate carboxykinase (*Pck1*) and glucose 6-phosphatase (*G6Pase*) compared with Control mice as shown in Fig. 3A and B.

To study whether there was a direct effect of E_2_/ESR1 on gluconeogenic genes, we examined the effect of E_2_ on gluconeogenic genes using primary hepatocytes. After 12 hours E_2_ treatment *ex vivo*, both *Pck1* and *G6Pase* mRNA levels were significantly inhibited by E_2_ (10^−12^ M, 10^−11^ M, 10^−10^ M, 10^−9^ M, 10^−8^ M) in a dose dependent manner (Fig. 3C,D). The IC50 for E_2_ inhibition of mRNA expression of *G6Pase* and *Pck1* was 1.006e^−10^ M, and 1.479e^−10^ M, respectively (Fig. 3C,D). Therefore, we focused our analysis using 10^−10^ M, 10^−9^ M, 10^−8^ M E_2_ in subsequent *ex vivo* studies. To determine whether ESR1 was necessary for the transcriptional repression of these genes elicited by E_2_, we isolated primary hepatocytes from Control mice and LERKO mice and examined the effect of E_2_ on gluconeogenic gene expression (*Pck1*, *G6Pase*) from these cells. As shown in Fig. 3E,F, the 10^−9^ M and 10^−8^ M doses of E_2_ significantly inhibited mRNA levels of *G6Pase*, while the 10^−8^ M dose of E_2_ significantly inhibited mRNA levels of *Pck1*. Importantly, this inhibition was completely abolished in primary hepatocytes from LERKO mice. The results demonstrated that E_2_ signaling has a direct inhibitory effect on gluconeogenic genes, and ESR1 was required for gene repression since the E_2_-elicited repression was completely abrogated in hepatocytes from LERKO mice (Fig. 3E,F).

To explore whether ESR1 binding to the promoter of gluconeogenic genes is altered by E_2_, we performed ChIP assay on *G6Pase* and *Pck1* promoters with or without E_2_. Putative consensus ESR1 binding sites were identified in the promoters of *G6Pase* and *Pck1* genes (ERE half site; AGGTCA)^30^. Immunoprecipitation of the chromatin collected from livers was performed following incubation with ESR1 antibody or IgG (negative control). Real-time quantitative PCR was then used to determine recruitment to the *G6Pase* or *Pck1* promoters. Our results demonstrated two-fold enrichment in the recruitment of the ESR1 to the *G6Pase* promoter relative to IgG (Fig. 3G) after E_2_ (10^−8^ M) treatment. Semiquantitative PCR products were compared in 1.5% agarose gels (Fig. 3G, right); Similarly, Fig. 3H showed about two-fold enrichment in the recruitment of the ESR1 to the *Pck1* promoter relative to IgG after E_2_ (10^−8^ M) treatment. Semiquantitative PCR products were compared in 1.5% agarose gels (Fig. 3G, right). As expected, basal recruitment of ESR1 to the *G6Pase* and *Pck1* promoters was nearly zero in primary hepatocytes from LERKO mice (Fig. 3G,H). In summary, ESR1 can be recruited to both *G6Pase* and *Pck1* promoters after E_2_ treatment, which may lead to direct inhibition of *G6Pase* and *Pck1* transcription observed in Fig. 3A and B.

### Effects of ESR1 on lipid metabolism

**Figure 4:**
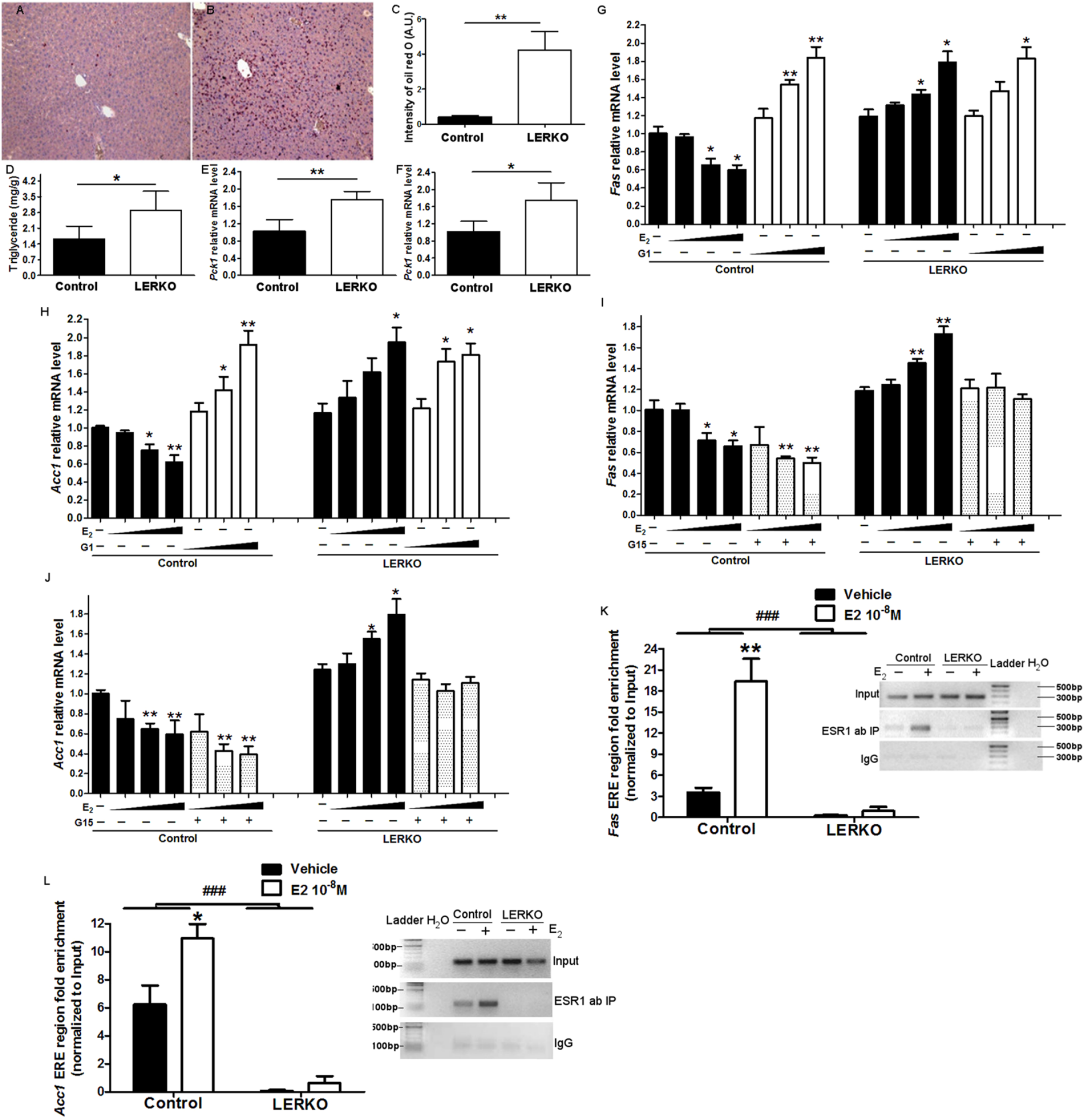
E_2_/ESR1 signaling inhibits hepatic lipogenesis. Representative images of liver sections from Control (**A**) and LERKO (**B**) mice after staining with Oil Red O as a measure of lipid accumulation (magnification: ×20). *n* = 3 per group. (**C**) Quantification of Oil Red O staining using Image (**J**). (**D**) Hepatic triglyceride levels in Control and LERKO mice. (**E**,**F**) mRNA levels of hepatic lipogenic genes *Fas* and *Acc1* in Control and LERKO mice were measured by q-RT-PCR. (**C**–**F**) *p < 0.05 versus Control, **p < 0.01 versus Control. Data from Fig. 4E,F are representative of results obtained from 6–10 mice in each group. (**G**,**H**). Primary hepatocytes from Control and LERKO mice were treated with vehicle or different doses of E_2_ (10^−10^ M, 10^−9^ M, 10^−8^ M) or GPER agonist G-1 (10^−10^ M, 10^−9^ M, 10^−8^ M), mRNA levels of *Fas* and *Acc1* were measured by q-RT-PCR. *p < 0.05 versus vehicle, **p < 0.01 versus vehicle, ***p < 0.001 versus vehicle. (**I**,**J**) Primary hepatocytes from Control and LERKO mice were treated with vehicle or different doses of E_2_ (10^−10^ M, 10^−9^ M, 10^−8^ M) in absence or in presence of GPER antagonist G-15 (10^−8^ M), mRNA levels of *Fas* and *Acc1* were measured by q-RT-PCR. *p < 0.05 versus vehicle, **p < 0.01 versus vehicle, ***p < 0.001 versus vehicle. (**K**,**L**) ChIP assay experiments were performed with liver tissues using antibody to ESR1, or with rabbit preimmune serum (IgG) and primers flanking the *Fas* (**K**) and *Acc1* (**L**) promoters. Real-time PCR data with an inset of a 1.5% agarose gel as a representative example. Results were normalized to input and shown as fold enrichment IgG from 3 independent ChIP experiments. The experiments were performed 2 weeks after virus injection. Values are means ± SD. *p < 0.05 versus IgG, ***p < 0.001 versus IgG.

To determine the effect of ESR1 KO on lipid metabolism, we performed Oil Red O staining of liver sections from Control and LERKO mice. Lipid accumulation was markedly more pronounced in LERKO mice (Fig. 4B) relative to Control mice (Fig. 4A). Quantification using ImageJ showed a significant increase in the intensity of Oil Red O staining in LERKO mice compared to Control mice (Fig. 4C) indicating LERKO mice accumulated more lipid droplets in the liver than Control mice. To further confirm this observation, total liver TG content was extracted as described^31^, and measured using the GPO-DAOS method. Hepatic TG content significantly increased by 55% in LERKO mice relative to Controls (Fig. 4D), confirming that there was increased lipid accumulation in the liver of LERKO mice.

### E_2_ inhibits *de novo* lipogenic genes through ESR1

*Fas* and *Acc1* are two critical lipogenic enzymes that catalyze the synthesis of fatty acids from acetyl-CoA. To determine whether increased expression of these genes may play a role in increased lipid deposition in livers of LERKO mice, hepatic mRNA levels of *Fas* and *Acc1* were assessed by q-RT-PCR. Significantly elevated hepatic mRNA levels of *Fas* and *Acc1* were observed in LERKO mice compared to Control mice (Fig. 4E,F).

To explore whether ESR1 exhibits direct inhibition of *Fas* and *Acc1* transcription, primary hepatocytes from Control and LERKO mice were isolated and treated with different doses of E_2_ (10^−10^ M, 10^−9^ M and 10^−8^ M), and *Fas* and *Acc1* mRNA levels were measured by q-RT-PCR. After E_2_ treatment, hepatocytes from the Control group exhibited a significant decrease in *Fas* and *Acc1* mRNA levels (Fig. 4G,H). Surprisingly, hepatocytes from LERKO mice showed elevation of both *Fas* and *Acc1* mRNA levels after E_2_ treatment (Fig. 4G,H). These results suggested that E_2_ inhibits *Fas* and *Acc1* mRNA levels in the presence of ESR1 as shown in Control mice, while E_2_ activates *Fas* and *Acc1* transcription in the absence of ESR1 as shown in LERKO mice. There is negligible expression of ESR2 in the liver (data not shown), however, GPER expression had been reported^32^. We hypothesized that GPER may mediate the effects of E_2_ on *Fas* and *Acc1* expression in the absence of ESR1. Primary hepatocytes from Control and LERKO mice were treated with different doses of GPER agonist G1 (10^−10^ M, 10^−9^ M and 10^−8^ M). G1 significantly increased *Fas* and *Acc1* mRNA levels in a dose dependent manner in both Control and LERKO mice (Fig. 4G,H). The effect of G1 on *Fas* and *Acc1* transcription in Control mice was similar to that of E_2_ in LERKO mice. These data suggested that GPER activates *Fas* and *Acc1* transcription. To further confirm that elevated expression of lipogenic genes in LERKO mice may result from activation of GPER in response to E_2_ in the absence of ESR1, we treated isolated primary hepatocytes from Control mice and LERKO mice with or without GPER antagonist G15 (10^−8^ M) in the presence of E_2_ (10^−10^ M, 10^−9^ M and 10^−8^ M), and measured mRNA levels of *Fas* and *Acc1* (Fig. 4I,J). The E_2_ elicited increase of mRNA levels of lipogenic genes was abrogated after G15 treatment in the LERKO group, suggesting that the increase of mRNA levels of *Fas* and *Acc1* was mediated by GPER, and that GPER plays a predominant role in *Fas* and *Acc1* transcription in the LERKO mice. Consistent with this observation, GPER mRNA level in LERKO mice was significantly increased relative to Control mice (Figure S1). Interestingly, the mRNA levels of *Fas* and *Acc1* were reduced by G15 in hepatocytes from Control mice (Fig. 4I and J), suggesting that even in the hepatocytes expressing ESR1, there was some stimulatory activity via GPER.

We next investigated whether the inhibition of *Fas* and *Acc1* mRNA involves recruitment of the ESR1 to the *Fas* and *Acc1* promoters after E_2_ treatment. We analyzed 5′ *Fas* and *Acc1* promoter sequences, as above, in order to identify putative ESR1 binding sites. We performed ChIP assay on ERE containing regions of the *Fas* and *Acc1* promoters with or without E_2_ treatment. As above, we immunoprecipitated the chromatin collected from primary hepatocytes of Control and LERKO mice with ESR1 antibody or IgG (negative control) and then performed real-time quantitative PCR to determine ESR1 recruitment to the *Fas* or *Acc1* promoters after E_2_ treatment. Our results demonstrated a six-fold and a nearly two-fold enrichment in the recruitment of the ESR1 to the *Fas* and *Acc1* promoters after E_2_ treatment, respectively (Fig. 4K,L). In summary, these studies demonstrated that E_2_ increased ESR1 recruitment to both *Fas* and *Acc1* promoters, which may lead to direct inhibition of *Fas* and *Acc1* transcription observed in Fig. 4E and F.

To understand the transcriptional control of overall lipid metabolism, we determined the expression of fatty acid oxidative genes. Fatty acid oxidative genes *Acot1*, *Acot2*, *Acsl1*, *Cpt2*, *Acox1*, *Hadh*, *Mcad and Lcad* were not changed in the liver of LERKO mice relative to Control mice (Figure S2). However, *Cpt1α*, a key mitochondrial enzyme for β-oxidation, was significantly elevated in LERKO mice (Figure S2), suggesting decreased lipid metabolism in LERKO mice.

### Effects of ESR1 on body weight and energy balance

**Figure 5:**
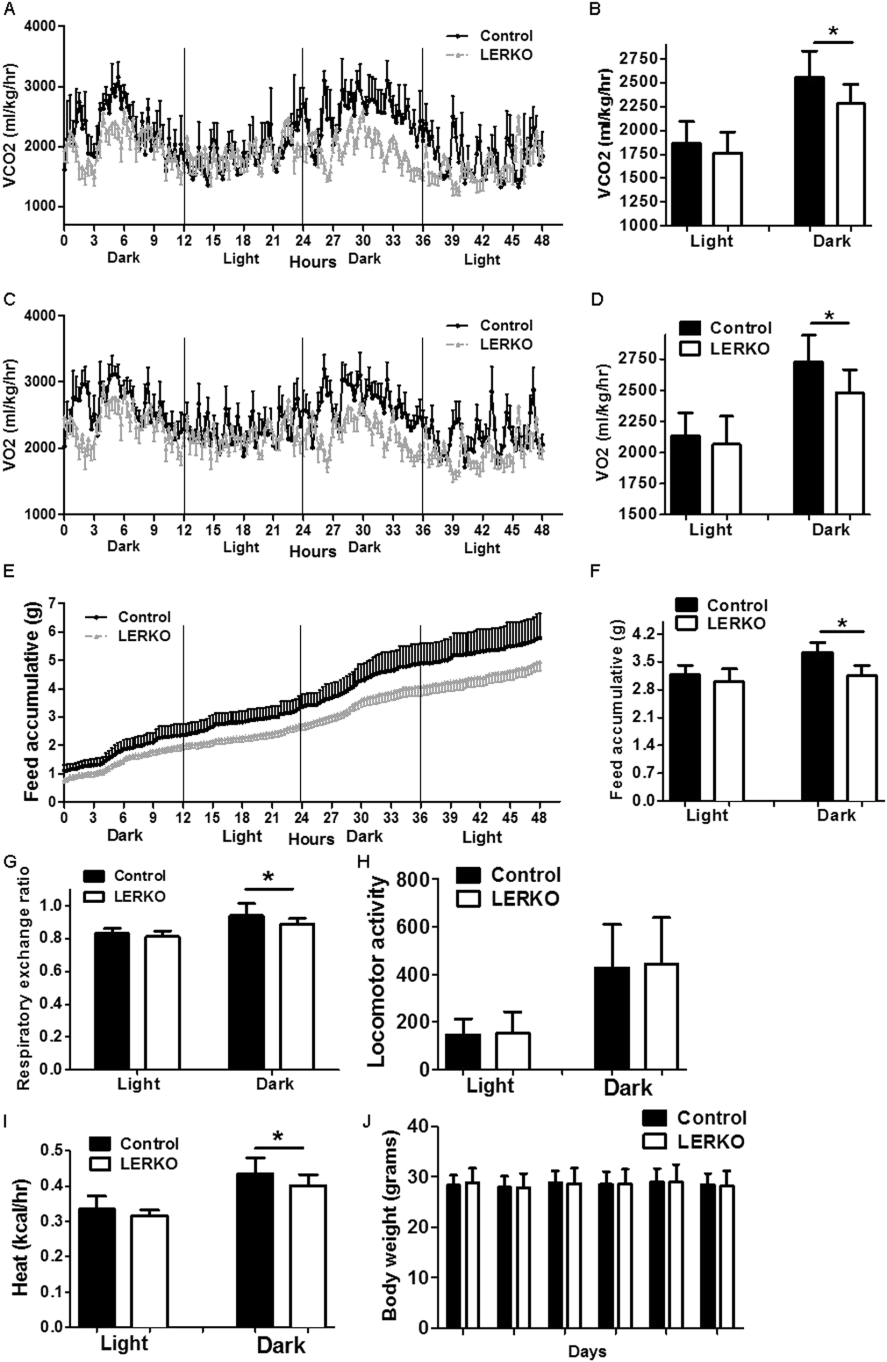
Reduced energy expenditure in LERKO mice. (**A**) Real-time monitoring curve of carbon dioxide release (VCO2). (**B**) Quantification of carbon dioxide release. (**C**) Real-time monitoring curve of oxygen consumption (VO2). (**D**) Quantification of O2 consumption. (**E**) Real-time monitoring curve of accumulated food intake. (**F**) Quantification of food intake. G, Respiratory exchange ratio (RER = VCO2/VO2) (**H**), Calculated body heat. (**I**) Locomotor activity. (**J**) Body weight measurement of Control group and LERKO group on GFP or CRE virus injection day 0, 4, 8, 12, 16. The experiments were performed 3 weeks after virus injection. Values are means ± SD. *p < 0.05.

In order to examine the impact of hepatic ESR1 on whole body energy expenditure and other metabolic parameters, we performed indirect calorimetry analyses on Control and LERKO mice using the CLAMS. Real-time monitoring showed VCO2 was decreased in LERKO mice compared to Control mice (Fig. 5A). Data from Fig. 5A is separated into dark and light cycle and is shown in Fig. 5B, and confirms LERKO mice exhibited a significant reduction in CO2 production. The reduction of VCO2 in LERKO mice relative to Controls was more pronounced in the dark cycle than the light cycle. Similarly, VCO2 and VO2 significantly decreased in LERKO mice relative to Control mice (Fig. 5C and D). These data indicate a reduced metabolic rate and decreased energy expenditure after hepatic *Esr1* knockout. The respiratory exchange ratio (RER) was calculated and was significantly decreased in LERKO mice relative to Control mice (Fig. 5G). Food intake was also assessed by the CLAMS and was significantly decreased in LERKO mice relative to Control mice (Fig. 5E) and quantified in Fig. 5F. Calculated body heat and general locomotor activity (Fig. 5H,I) were not changed significantly in LERKO mice.

To examine whether the difference observed in CLAMS were caused by change of body weight in LERKO mice, we weighed the mice for more than 2 weeks after virus injection. There was no significant difference in body weight between Control mice and LERKO mice (Fig. 5J), suggesting that metabolic and behavioral changes observed in LERKO mice are not indirectly attributable to changes in body weight.

## Discussion

Impaired estrogens signaling is linked to obesity, metabolic dysfunction and increased risk of chronic disease in both humans and in animal models^3, 33, 34^. Estrogens deficiency contributes to the development of obesity, hyperglycemia, and type 2 diabetes. Additionally, *Esr1* gene variants may reveal functionally important regions related to type 2 diabetes^19, 33, 35^. However, the mechanisms underlying these findings have yet to be determined. In our current study we investigated the importance of hepatic ESR1 action on the manifestation of metabolic dysfunction and the mechanisms underlying these outcomes.

Our study reveals a role for hepatic ESR1 on lipid and glucose metabolism in normal male mice. Previous studies disrupting *Esr1* in the liver using a conditional knock-out paradigm (we will call dLERKO mouse) have produced conflicting results. Della Torre *et al*. identified a role for hepatic ESR1 in regulating IGF1 synthesis^23^ and reproductive function in female mice^24^. They also noted ESR1 regulation of the synthesis of cholesterol transport proteins, enzymes for lipoprotein remodeling, and receptors for cholesterol uptake^24^. In contrast, other groups have observed very different phenotypes including one study that showed no difference in metabolic function, growth or fertility^36^, and another that showed that the absence of hepatic ESR1 was associated with the development of fatty liver and hepatic insulin resistance during HFD feeding^21, 22^. These latter reports by the Stafford group only identified a difference in metabolic phenotype between Control and dLERKO mice fed a high fat diet without changes in glucose or lipid metabolism observed in chow fed male^22^ or female mice^21^. However, it is important to highlight that all of the aforementioned studies mated an albumin-CRE mouse with a floxed *Esr1* mouse to develop their dLERKO mice, therefore, any developmental effects of E_2_ signaling in the liver would be lost. We have used tail vein injections of CRE expressing AAV8 to acutely knockout *Esr1* in adult mice (LERKO mice). Our studies implicate ESR1 signaling in normal liver function in non-obese male mice. This reveals a role for hepatic ESR1 not observed in previous models of hepatic *Esr1* knockout, with the difference perhaps due to compensatory changes in liver function during embryonic and neonatal development.

Our results suggest hepatic ESR1 inhibits *Pck*-*1* and *G6Pase* genes through direct inhibition at the transcriptional level (Fig. 3A,B) and, therefore, ESR1 may play a protective role in glucose metabolism. Lundholm *et al*. have reported that the ESR1 agonist propyl pyrazole triol (PPT) improves glucose tolerance via depression of the *G6p* gene expression in female obese mice^37^. Consistent with this finding, upregulation of *Pck*-*1* and *G6Pase* gene expression was accompanied with downregulation of ESR1 (Figs 1 and 3A and B). E_2_ caused ESR1 to be recruited to the promoters of both the *Pck*-*1 and G6Pase* genes, suggesting ESR1 might directly bind to gluconeogenic genes and inhibit their transcription. However, this cannot exclude the possibility that this inhibition may be mediated by insulin signaling and/or be indirectly affected by ESR1.

Our study showed that ESR1 inhibition significantly affects lipid metabolism. FAS catalyzes the *de novo* synthesis of fatty acids, and it has been shown to play a key role in liver physiology through signaling as well as energy storage^38^. It increases stored and secreted hepatic triglycerides by increasing de novo synthesized lipids. In humans, on diets low in fat and high in carbohydrate (10% of calories as fat and 75% as carbohydrate), *de novo* lipogenesis makes a significant contribution to circulating lipids as almost half of VLDL triglyceride is derived from DNL under these conditions^39^. In our current study, LERKO mice demonstrated higher level of hepatic triglyceride as well as elevated *Fas* and *Acc1* expression, suggesting hepatic FAS and ACC1 increased lipid storage in the absence of ESR1. ESR1 binds to *Fas* and *Acc1* promoters in response to E_2_ treatment as measured by ChIP assay (Fig. 4K,L) suggesting direct E_2_/ESR1 inhibition of *Fas* and *Acc1* expression, which is lost when ESR1 is disrupted in LERKO mice.

Interestingly, while E_2_ down regulated expression of *Fas* and *Acc1* in Control hepatocytes, E_2_ upregulated expression of these genes in LERKO hepatocytes. This result suggested that other receptors might be involved in E_2_ mediated *Fas* and *Acc1* transcriptional activation. ESR2 mRNA levels were barely detectable and not different in the LERKO mice relative to the Control mice (data not shown), suggesting ESR2 does not play a role in the E_2_ regulation of lipogenic genes. GPER is a recently described estrogen receptor^40^ that can bind to E_2_ and activate cytoplasmic signal transduction pathways in response to E_2_. While GPER does not exhibit estrogenic responses in reproductive organs in mice^41^, it has been shown to play a key role in the regulation of insulin levels and glucose tolerance^42^, suggesting its importance in metabolism. Other reports describe contrasting effects of ESR1 and GPER in cell proliferation^43^ and dopamine transporter function^44^ with these two estrogen receptors appearing to balance each other. So in the absence of ESR1, GPER is increased (Figure S1) and be activated in the liver of LERKO mice and mediate E_2_ induced activation of *Fas* and *Acc1* transcription.

In our current study, the presence of fatty liver is accompanied with increased gluconeogenesis. This finding is consistent with clinical data. Approximately 20% of the U.S. population has nonalcoholic fatty liver disease (NAFLD), about 75 to 100 million people in the United States are affected^45^, and this disease has been associated with disrupted glucose homeostasis. One possible mechanism for contributing to both increased hepatic lipogenesis and gluconeogenesis is impaired insulin signaling. Patients with NAFLD showed impaired glucose metabolism associated with impaired insulin sensitivity^46, 47^. Increased hepatic lipid deposition has been implicated in lipid-induced increases in liver diacylglycerol (DAG) content, leading to activation of PKC-θ^48^ or –ε^26^ and subsequent decreased insulin signaling^26^ and impaired gluconeogenesis. Therefore, the increase in hepatic lipid content in the LERKO mice could indirectly result in increased gluconeogenesis. Identifying whether there is a connection between dysfunction of lipid metabolism and glucose metabolism in our model still requires further investigation.

E_2_/ESR1 regulation of energy homeostasis has diverse effects on different tissues. Hypothalamic POMC neuron-specific ESR1 deletion impairs fertility but has no effect on energy expenditure, while deletion of ESR1 in hypothalamic steroidogenic factor-1 neurons resulted in both infertility and decreased energy expenditure and metabolism in females^49^. At the level of the muscle, in female mice, ESR1 serves to prevent retention of dysfunctional mitochondria and muscle-specific *Esr1* knock out results in impaired glucose metabolism and increased adiposity^50^. Less is reported on the role of ESR1 in males. In our study, we observed a reduced energy expenditure in LERKO mice, suggesting a trend of hypometabolism. The mechanism by which ESR1 regulates energy expenditure, however, is unknown, but could include changes in mitochondrial function as described by others^50, 51^. No weight difference was observed despite the decreased energy expenditure, likely because of a decrease in food intake in LERKO mice compared to Control mice. The respiratory exchange ratio (RER) was found to be decreased in LERKO mice, suggesting a moderate increase in the use of fat as an energy substrate, possibly as a consequence of the increase in *de novo* lipogenesis by the liver.

The results of this study provide several novel insights regarding the role of hepatic ESR1 in glucose and lipid metabolism in male mice including the observation that ESR1 inhibits gluconeogenesis by transcriptional inhibition of gluconeogenic genes *Pck*-*1* and *G6Pase*. Additionally, ESR1 plays a role in normal hepatic lipid metabolism, possibly by direct transcriptional regulation of expression of key genes in DNL including *Fas and Acc1*.

Taken together, these data support a model in which hepatic ESR1 plays a protective role in glucose and lipid metabolism. Impaired hepatic E_2_ action in male mice, produced by hepatic specific *Esr1* ablation, could recapitulate aspects of the metabolic syndrome. This suggests the ESR1 signaling pathway can be a relevant therapeutic target to prevent and reverse lipid and glucose metabolic dysfunction.

## Methods

### Animals

All animal studies were carried out in accordance with National Institutes of Health guidelines on animal care regulations and were approved by the Animal Care and Use Committee of the Johns Hopkins University. We generated floxed ESR1 mice as previously described^52^. ESR1 floxed mice were treated via tail-vein injection with adeno-associated virus expressing bacteriophage CRE recombinase under the liver-specific thyroid binding globulin promoter (AAV8. TBG-CRE) to conditionally ablate ESR1 specifically in liver at the age of 2–3 months. Control mice received an AAV8 expressing green fluorescent protein (GFP; AAV8-TBG-GFP) at the age of 2–3 months (Both vectors were from Penn Vector Core, University of Pennsylvania). All *in vivo* experiments were performed at least one week after virus injection. Viral efficacy was confirmed with equivalent *Esr1* knock down by AAV8-CRE for at least 4 months following injection (data not shown).

### Intraperitoneal Glucose Tolerance Test/Pyruvate Challenge Test/Insulin Tolerance Test

IPGTT: mice were fasted 16 hours and then injected with 2 mg of glucose/kg body weight intraperitoneally. IPPCT: mice were fasted 6 hours and injected with 2 mg of pyruvate/kg body weight intraperitoneally. IPITT: mice were fasted 7 hours and injected with 0.3 unit of insulin/g body weight intraperitoneally. Blood glucose was obtained from tails and blood glucose levels were determined using a blood glucose meter (OneTouch Ultra, LifeScan). See supplementary material for detailed information.

### Primary hepatocyte cultures

Primary hepatocyte culture experiments were performed three weeks after virus injection. Primary hepatocytes were isolated from livers (Supplementary material), and plated at 0.8 × 10^6^ cells per well of six-well dish in Williams E supplemented with 10% FBS (Gibco). Four to six hours after plating, hepatocytes were treated with different doses of E_2_ (ranging from 10^−12^ M–10^−8^ M) for 12 hours.

### Glucose production assay

Mouse primary hepatocytes were cultured in six-well plates with William’s medium E supplemented with ITS (BD Biosciences) and dexamethasone (10 nM). After 4–6 hours *in vitro* incubation, cells were treated with E_2_ (10^−8^ M) or vehicle for 8 hours. Glucose release from hepatocytes was determined during incubation in glucose-free DMEM supplemented with sodium lactate (20 mM) and sodium pyruvate (2 mM) with or without E_2_ (10^−8^ M). Glucose output into the culture medium over 4 hours was measured enzymatically using glucose oxidase (EnzyChrom, BioAssay Systems, CA), normalized to cellular protein content (BCA method) Glucose production was expressed as nmol of glucose produced/hour/total protein.

### Tissue fatty acid composition and serum chemistry

Mice were fasted overnight, and serum was collected by facial vein puncture. Serum insulin was measured on a plate based assay (Luminex 200 Millipore (Billerica, MA). Lipids were extracted from 50 mg flash-frozen liver from Control and LERKO mice as described^31^. Triglyceride levels were measured in extracted lipids by GPO (glycerol-3-phosphate oxidase) -DAOS (N-ethyl-N-(2-hydroxy-3-sulfopropyl)-3,5-dimethoxyaniline sodium salt) method (LabAssay^TM^ Triglyceride kit, Wako Biochemical, Richmond, VA).

### Histology and Immunofluorescence

Liver tissue was fixed in 4% paraformaldehyde for 4 hours, immersed in 30% sucrose, embedded in Tissue-Tek O.C.T (optimum cutting temperature) compound and sectioned (5 um) before immunofluorescence staining, haemotoxylin and eosin staining, or Oil Red O staining. Images were obtained with a microscope equipped with a digital color camera (Leica, DM4000, Germany).

For immunofluorescence, tissues were treated with 1% Triton and blocked in 5% goat serum, and then exposed to anti-ESR1 rabbit (Millipore, California, CA) at a 1:400 dilution in 1% goat serum overnight at 4 °C. Slides were incubated with Alexa Fluor 594 goat anti-rabbit IgG (H + L) for 1 hour before nuclear counterstaining with DAPI and mounting (Vectashield Vector laboratories, Burlingame, CA). For ESR1 immunofluorescence quantification, 5 representative images were selected from the specimen and fluorescence intensity was analyzed using ImageJ (National Institute of Health).

### Q-RT-PCR

See supplementary material. Primer sequences for the selected genes are described in Table 1. Fatty acid oxidative genes primers are cited from^53^.

**Table 1:** Sequences of primers.

Gene name	Forward primer	Reverse primer
*G6Pase*	AAAAAGCCAACGTATGGATTCCG	CAGCAAGGTAGATCCGGGA
*Pck1*	AGCATTCAACGCCAGGTTC	CGAGTCTGTCAGTTCAATACCAA
*Fas*	GAGGACACTCAAGTGGCTGA	GTGAGGTTGCTGTCGTCTGT
*Acc1*	ATGGGCGGAATGGTCTCTTTC	TGGGGACCTTGTCTTCATCAT
*Cpt1α*	CCATCCTGTCCTGACAAGGTTTAG	CCTCACTTCTGTTACAGCTAGCAC
*Cpt2*	CAACTCGTATACCCAAACCCAGTC	GTTCCCATCTTGATCGAGGACATC
*Mcad*	AACACTTACTATGCCTCGATTGCA	CCATAGCCTCCGAAAATCTGAA
*Lcad*	TTTCCTCGGAGCATGACATTTT	GCCAGCTTTTTCCCAGACCT
*Acot1*	GACAAGAAGAGCTTCATTCCCGTG	CATCAGCATAGAACTCGCTCTTCC
*Acot2*	AGTCAACGACGCAAAATGGTG	GCTCTTCCAATCCTGTTGGC
*Acsl1*	ATCTGGTGGAACGAGGCAAG	TCCTTTGGGGTTGCCTGTAG
*Hadh*	TGCATTTGCCGCAGCTTTAC	GTTGGCCCAGATTTCGTTCA
*Acox1*	ACGCCACTTCCTTGCTCTTC	AGATTGGTAGAAATTGCTGCAAA
*GPER*	GTCACGCCTACCCCTTGACA	CCTGAAGGTCTCTCCCAGGAA
*18s*	TGGTTGATCCTGCCAGTAG	CGACCAAAGGAACCATAACT
**ChIP Primers**	**Forward primer**	**Reverse primer**
*G6Pase* ERE	TAAATATTTTTATCTCATGTGCATTGG	CACTGGATGGTCTTCAAGAGG
*Pck1* ERE	CAACAGGCAGGGTCAAAGTT	GCACGGTTTGGAACTGACTT
*Fas* ERE	GCACGGCCCAGACTCTGCAT	GCTTGCCCCCAAGCTCTCCC
*Acc1* ERE	ACAGGAGGTAATGACAGGGGAGGG	CGTGCGTGCGTGTGTGTGTG

### Chromatin immunoprecipitation analysis

Primary hepatocytes from Control and LERKO mice were prepared and subjected to chromatin immunoprecipitation using the ChIP-IT Express kit (Active Motif, Carlsbad, CA; supplementary material). Primers that encompassed the mouse *G6Pase*, *Pck1*, *Fas* and *Acc1* promoters regions (to assess binding to putative ERE-containing regions) were used for q-RT-PCR (Table 1). *Pck1* primer sequences were obtained from^54^.

### Indirect Calorimetry

Mice were allowed to acclimate to respiratory chambers for one day. Subsequently, oxygen consumption, carbon dioxide production, respiratory exchange rate, heat, activity and food intake were measured for 48 hours during 12-h light/12-dark cycles using Comprehensive Lab Animal Monitoring System (CLAMS) (Columbus Instruments, Columbus, OH).

### Statistical Analyses

All data are reported as means ± SD, with ‘n’ representing the number of mice or samples used in each of the experimental groups. Significance was determined using the two-tailed unpaired Student’s t-test or by ANOVA using GraphPad Prism 6.0 (post hoc by Bonferroni’s method). For all analyses, statistical significance was accepted at *p* < 0.05.

## Electronic supplementary material

Supplementary material
