# Supplementary material for: Hepatic estrogen receptor α is critical for regulation of gluconeogenesis and lipid metabolism in males
Source: Sci Rep. 2017 May 10;7:1661. doi: 10.1038/s41598-017-01937-4 (PMC5431852; doi:10.1038/s41598-017-01937-4)
Supplement: Supplementary file 1 — Supplementary material [file 41598_2017_1937_MOESM1_ESM.pdf]

Hepatic estrogen receptor α is critical for regulation of gluconeogenesis and lipid metabolism in males

Shuiqing Qiu[1], Juliana Torrens Vazquez[2], Erin Boulger[3], Haiyun Liu[4], Ping Xue[1], Mehboob Ali Hussain[1], Andrew Wolfe[1]

[1] Division of Metabolism and Pediatric Endocrinology, Departments of Medicine, Pediatrics, Biological Chemistry and Physiology, Johns Hopkins University School of Medicine, Baltimore, MD, USA.

[2] School of Medicine, Ponce Health Sciences University, Ponce, PR, USA.

[3]School of Engineering, Johns Hopkins University, Baltimore, MD, USA.

[4]Department of Dermatology, Johns Hopkins University School of Medicine, Baltimore MD, USA.

Corresponding author

Andrew Wolfe

600 North Wolfe Street, CMSC-406, Baltimore, Maryland, USA

Telephone number: 410-502-7916

Electronic address: awolfe3@jhmi.edu.

Supplementary material

Intraperitoneal Glucose Tolerance Test/ Pyruvate Challenge Test/ Insulin Tolerance Test

The blood was collected through the tail vein for glucose determinations at 0, 15, 30, 60, 90, 120 min. At the end of the glucose tolerance test or pyruvate challenge test, mice were returned to cages and allowed free access to food. Blood glucose levels were determined using a blood glucose meter (OneTouch Ultra, LifeScan).

Primary hepatocytes cultures

Primary hepatocytes were isolated from livers of 14-16-week-old control and LERKO mice maintained on a standard chow diet. Mice were anaesthetized with a ketamine/xylazine mixture. Abdominal cavity, liver, portal vein (PV), and inferior vena cava (IVC) were sufficiently exposed. PV was catheterized with a 24-gauge catheter (Becton Dickinson), and PBS containing 1mM EGTA and Pen/Strep (Gibco) infused into the liver for 5-6 min at 7ml/min followed by type IV collagenase (Sigma) for 9 min at the same flow rate. Liver cells were filtered and spun at 50 g to remove dead cells and non-hepatocytes. After three washes, cells were plated at 0.8×10^6 cells per well of six-well dish in Williams E supplemented with 10% FBS (Gibco). Four to six hours after plating, hepatocytes were treated with different doses of E_2 (ranging from 10^{-12}M- 10^{-8}M) for 12 hours.

Q-RT-PCR

The targeted mRNA levels were examined by quantitative RT-PCR using the CFX Connect™ Detection System (Bio-Rad). Total RNA was prepared using TRIzol reagent (Invitrogen), and cDNA was synthesized using the iScript cDNA Synthesis Kit (Bio-Rad). Relative cDNA levels were determined using SYBR Green master mix Solution (Bio-Rad).

Chromatin immunoprecipitation analysis

After 3 hours of steroid deprivation followed by 45 min of E_2 (10^{-8}M) treatment, primary hepatocytes from control and LERKO mice were fixed in 1% formaldehyde, and the fixation was stopped by glycine. The homogenate was centrifuged at 1000 rpm for 5 min at 4°C. The supernatant was discarded, and the nuclear pellet was resuspended in nuclear lysis buffer and incubated on ice for 30 min. Dounce homogenization aided in nuclei release. Nuclear samples were sonicated on ice for 5 18-sec cycles with 1-min pauses between each cycle using Branson digital sonifier Model 250&450 (Danbury, Connecticut) at 10% amplitude. After sonication, the samples were centrifuged at 15000 rpm for 10 min at 4°C. The supernatants were then collected and stored at -80°C. Primers that encompassed the mouse *G6Pase*, *Pck1*, *Fas* and *Acc1* promoters regions (spanning putative ERE-containing regions) were used for q-RT-PCR.

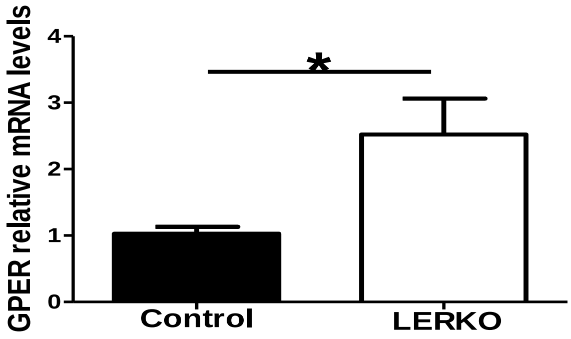

Figure S1.mRNA level of *Gper* in Control and LERKO mice were assessed by q-RT-PCR. * $p<0.05$ versus Control. Data is representative of result obtained from 7 mice in each group. The data are expressed as the means±SD, *$p<0.05$, Control versus LERKO.

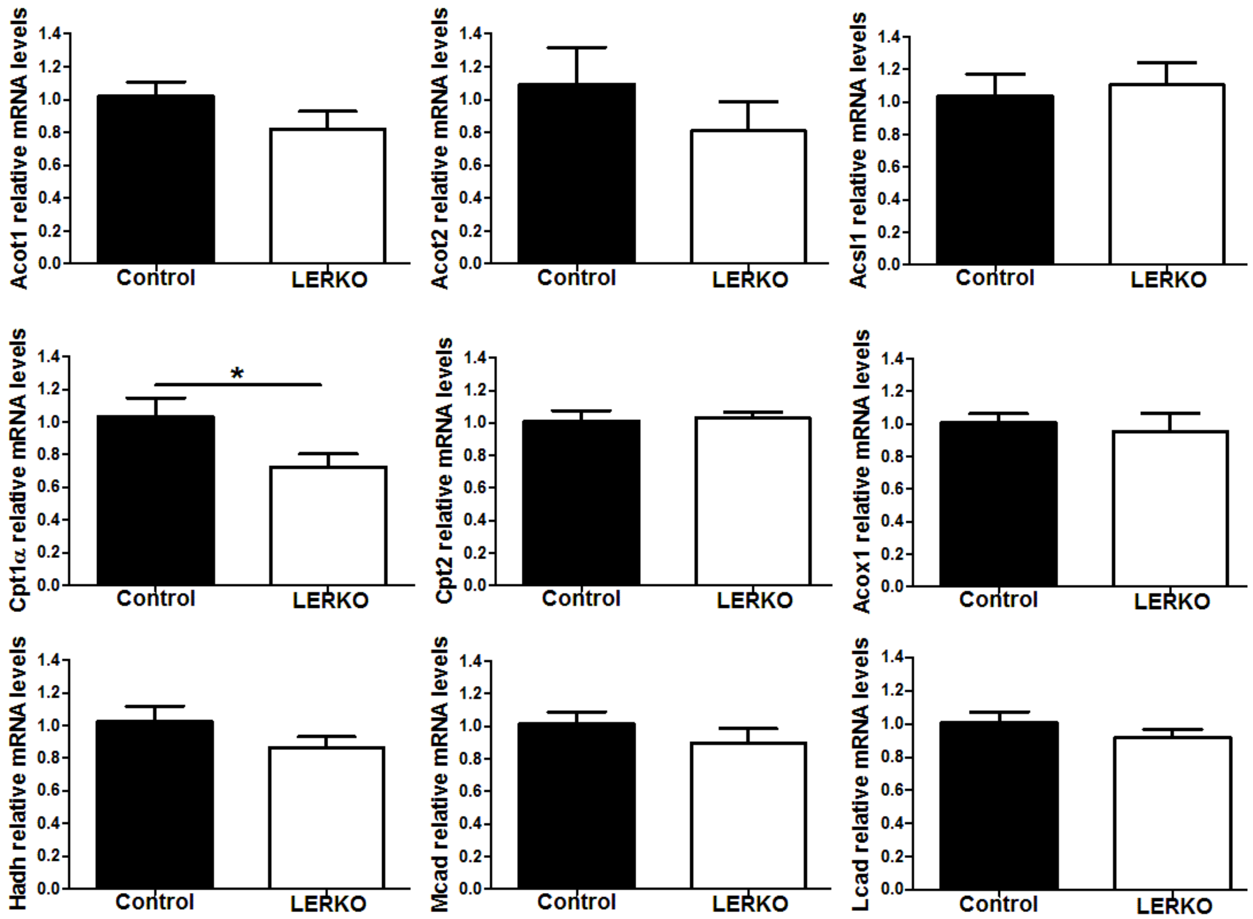

Figure S2. Fatty acid oxidative genes mRNA levels were measured by q-RT-PCR. A-I, Hepatic mRNA levels of *Acot1*, *Acot2*, *Acsl1*, *Cpt1α*, *Cpt2*, *Acox1*, *Hadh*, *Mcad*, *Lcad* from Control mice and LERKO mice (n=7 per genotype). The experiments were performed 4 weeks after virus injection. The data are expressed as the means±SD, *p<0.05, Control versus LERKO.
